# Changes in Baropodometric Evaluation and Discomfort during the Workday in Assembly-Line Workers

**DOI:** 10.3390/healthcare12070761

**Published:** 2024-03-31

**Authors:** Juan Rabal-Pelay, Cristina Cimarras-Otal, Belén Lacárcel-Tejero, Andrés Alcázar-Crevillén, José Antonio Villalba-Ruete, César Berzosa, Ana Vanessa Bataller-Cervero

**Affiliations:** 1 ValorA Research Group, Universidad San Jorge, Autovía A-23 Zaragoza-Huesca km 299, 50830 Villanueva de Gállego, Spain; jrabal@usj.es (J.R.-P.); cberzosa@usj.es (C.B.); avbataller@usj.es (A.V.B.-C.); 2 Hospital MAZ, Avda. Academia General Militar, 74, 50015 Zaragoza, Spain; 3 BSH Electrodomésticos España S.A., Polígono Industrial La Cartuja Baja, Ctra. Castellón, km 6.300, Cartuja Baja, 50720 Zaragoza, Spain

**Keywords:** foot pressure, lower-back discomfort, work safety, lower-limb discomfort, manufacturing company

## Abstract

Prolonged standing at work is associated with health risks. The appearance of lower-limb and lower-back discomfort is one of the most prevalent factors in prolonged standing workers. The aim of this research was to evaluate the effect of an eight-hour workday on foot pressure and musculoskeletal discomfort in standing workers. Thirty-six assembly-line workers (six women) were recruited to participate in a cross-sectional study to assess foot pressure and surface, foot, knee, and lower-back discomfort before and after a real workday. Baropodometry outcomes (surface and pressure) were evaluated by the pressure platform SensorMedica and musculoskeletal discomfort was evaluated by Cornell’s Musculoskeletal Discomfort Questionaire. Total foot surface (*p* = 0.01) and foot discomfort (*p* = 0.03) increased significantly at the end of the workday. Prolonged standing during 8 h workday increased the foot discomfort and total foot surface in assembly-line workers. No foot pressure variable (forefoot, rearfoot, or total) was significantly modified after the workday in assembly-line workers.

## 1. Introduction

Work-related musculoskeletal disorders (MSD) are the most prevalent occupational disease in the world [1,2]. Millions of European workers were affected by this cause in every employment sector, regardless of the type of work sector. Figures shown by Eurostat [3] quantify MSD with 39% of the occupational diseases that cause a large number of sick days every year. Among the risk factors for MSD are excessive task repetitions, heavy load lifting, and awkward and static postures [4]. MSD causes loss of productivity and work disability [5,6]. The manufacturing industry is also affected by this kind of sick leave [7]. In manufacturing, workers on the assembly lines spend most of their working time in prolonged standing or sitting positions, making repetitive movements with upper limbs [8].

Standing work performed by manufacturing workers is associated with lower-limb and trunk discomfort [9,10,11,12] and vascular disorders [13,14,15,16]. Prolonged standing has been identified as a risk factor for lower-back pain and discomfort [12,17]. Lower-limb pain is highly prevalent among the European workforce, and work exposures are a major contributing factor [18]. The Canadian Centre for Occupational Health and Safety (CCOHS) [19] has reported back pain, sore feet, varicose veins, general muscular fatigue, and other health problems related to prolonged standing work. Moreover, the spine, hips, knees, and ankles can become locked for periods of time, which could lead to rheumatic problems [20]. Prolonged static posture can lead to muscle ischemia, trigger points, joint hypomobility, pain, and protective muscle contraction in order to prevent pain [21]. The stress suffered by the lower limbs and spine during standing work depends on such factors as body mass, load, and the duration of standing [22]. Not only could the standing posture present a risk of producing discomfort in assembly-line workers, but also wearing safety shoes could generate discomfort in the workers [23], modifying kinematic responses [24,25] or even involving a risk of suffering musculoskeletal injuries [26]. Using padded or customized insoles is one of the strategies to mitigate pressure on the sole of the foot when wearing safety shoes [24,27,28].

The measurement of the pressure distribution in the foot is considered a method to characterize foot function [29]. Plantar pressures have been related to foot pathologies [30]. Several studies have identified an association between foot/leg pain and high pressure in the metatarsal region [4]. However, the cause–effect relationship is not clear.

Plantar pressure evaluation may result in interest in understanding the effects of the load on the foot and the effect of the work-related discomfort. The load effect could cause discomfort in the lower limb or other body regions, such as the spine. Foot pressure and spinal posture seem to be interrelated under static and dynamic conditions [31].

In workers wearing safety shoes, normal and fast walking were found to be the most demanding activities in terms of peak pressure [24]. Wearing safety shoes results in high pedobarographic parameters in several foot regions. It also modifies biomechanical parameters and occupational task performance with respect to normal footwear [32,33]. Discomfort caused by walking also modifies the foot pressure pattern, showing more heel loading [34]. It has been observed in standing workers that there was an increase in the distribution of plantar pressure and the perception of fatigue in the lower extremities after a five-hour shift [35]. It was found that the workload of production workers increased the perception of fatigue after 3 h of work without modifying the workers’ plantar pressure [36].

The aim of the research was to analyze foot surface, pressure, and perception of discomfort in the back and lower extremities (knees and feet) in assembly-line workers pre- and post- an eight-hour workday in real work conditions of a manufacturing company. In addition, the possible association between changes in foot mean pressure and perceived discomfort was studied. The hypothesis of the work is that the total foot surface, as well as the discomfort in the lower limbs, will increase at the end of the workday.

## 2. Materials and Methods

The present investigation is a descriptive cross-sectional study that evaluates the effect of an eight-hour workday on the perception of discomfort, surface area, and pressure of the feet.

### 2.1. Participants

Thirty-six volunteer workers (30 male, 6 female) were recruited for this study. All of them were full-time workers on the assembly line in a manufacturing company. They were recruited by the company’s medical service. All participants had a minimum of two years of experience in the company. In the assembly line, the workers assembled electrical appliances, having to carry out work of screwing, placing parts, and lifting light weights. The space they had to work was 2 m wide, and they did not have to walk to obtain the components or tools. The assembly line is at the same height for everyone. All the workers wore the same brand of iron-toed safety shoes. The workday started at 6 and ended at 14:00. The work task was changed every two hours. Workers had twenty-five minutes in the middle of the workday to take a break and have a drink/eat.

The inclusion criteria were to work for 8 h in a standing position in an assembly line. Exclusion criteria were not having had spine or feet surgery, being diagnosed with scoliosis, wearing custom insoles, being pregnant, or/and having been on sick leave in the three months prior to this study. Participants who had worn customized insoles throughout their lives for the treatment of any condition were excluded. All the participants were informed about the procedure of this study and gave informed consent. This study was conducted in accordance with the Declaration of Helsinki of 1961, and the protocol was approved by the committee of ethics in research of the government of the region (Cómite de Ética de la Comunidad de Aragón) [C.I. PI16/0140].

### 2.2. Outcomes Assessment

Assessments were evaluated in the medical service of the manufacturing company. All measurements were performed by the same researcher. The researcher who carried out the assessments was different from the one who carried out the statistical analysis. All assessments were taken on Monday to avoid over-week fatigue. The measurements were taken at the beginning of the workday, at 6 o’clock in the morning, and repeated at 14 o’clock in the afternoon, at the end of the workday.

The workers were measured in underwear and without socks to measure their height, weight, and plantar pressure. Data were collected as previously described in Rabal-Pelay et al. (2019) [37]. Specifically, the following measurements were taken:

Height: The height (cm) of participants was measured using a SECA^®^ stadiometer (model 206, Seca Corp., Hanover, MD, USA) with a precision of 1 mm and a range of 130–210 cm, according to the International Society for the Advancement of Kinanthropometry (ISAK) standards [38]. Participants were measured with their backs in contact with the wall and facing forward in the Frankfort position.

Body mass: Body mass was measured in underwear with a SECA^®^ scale (model 799, Seca Corp., Hanover, MD, USA), with a precision of 0.1 kg and a range of 2 kg–200 kg. 

**Figure 1:**
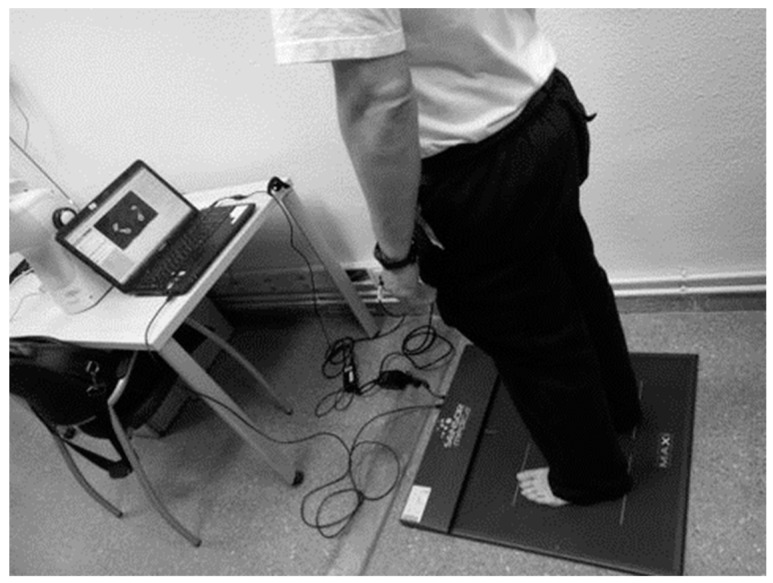
Example of baropodometric outcomes assessment.

Plantar pressure: Foot pressure distribution was measured with a Freemed baropodometric pressure platform (SensorMedica, Rome, Italy), composed of a pressure-sensitive plate with an active surface of 400 mm × 400 mm and 8 mm thick. The platform weighs 4 kg and contains resistive sensors, gold-coated 24k with conductive rubber. The number of sensors per square meter is 10.000, and the platform supports a maximum pressure of 150 N/cm^2^. Acquisition frequency 400 Hz. FreeStep v.1.0.3 software (Rome, Italy) was used to record and analyze the data. The reliability of this baropodometric platform has been shown in other studies [39]. The subject was asked to stay for 10 s as still as possible in a barefoot standing position, with feet approximately at pelvis width, looking straight ahead and keeping their arms at their sides in a comfortable position (anatomically neutral posture). The measurement was performed twice for each participant (Figure 1). For each baropodometry register, the surface (cm^2^) and mean pressure (kPa) of each foot were analyzed. Moreover, to analyze the pressure by region, the foot was divided into forefoot and rearfoot regions, taking the mean pressure (kPa) in each region. Forefoot and rearfoot variables correspond to the plantar pressure sum (kPa) of the right and left feet. Left and right mean pressure correspond to the plantar pressure of each separate foot. Total foot pressure corresponds to the sum of the right and left feet. Total foot pressure corresponds to the sum of the right and left feet. The total foot surface corresponds to the sum of the right and left feet. The mean pressure was established from the 10-s recording. For defining the fore and rear foot areas, the software divides the footprint, considering 40% of the surface for the forefoot and the remaining 60% of the surface for the rearfoot. The division percentages are made on the length of the foot.

Back and lower-limb discomfort: Discomfort was assessed using the Cornell Musculoskeletal Discomfort Questionnaire (CMDQ) for standing workers. The questionnaire evaluates the frequency, discomfort, and interference caused by discomfort of each body area. In this research, only the lower-back and lower-limb (knees and feet) regions were registered. The discomfort total score for each body region studied was calculated by multiplying discomfort severity (0, 1, 2, 3 values) and work interference score (with values of 1, 2, or 3). For feet and knees, the sum of the discomfort indicated in the right and left limbs has been considered for the analysis. This questionnaire has been validated for the Spanish population [40].

### 2.3. Statistical Analysis

The normality of the variables was assessed by the Kolmogorov–Smirnov test. A Mann–Whitney U test was used for a gender comparation of baseline characteristics at the beginning of the workday. A *t*-test of independent samples was analyzed to study the anthropometric and plantar pressure differences between men and women. A *t*-test of related samples was applied to analyze the differences in plantar pressure variables between pre- and post-work evaluations. Wilcoxon signed-rank test was performed for discomfort variables, which did not have a normal distribution. The effect size between the pre- and post-work evaluations was analyzed with Cohen’s d statistic. It was defined that d of Cohen values lower than 0.20 indicated the absence of an effect; values between 0.21 to 0.49 indicated a small effect; values between 0.50 and 0.70 indicated a moderate effect, and values greater than 0.80 indicated a large effect [41]. Effect size values were reported with 95% confidence limits.

The correlation between the studied variables was analyzed using Pearson and Spearman correlation coefficients. Pearson’s correlation was used in the case of normally distributed variables (anthropometrics and plantar pressure), while Spearman correlation was used to correlate variables with non-normal distribution (discomfort). Cut-off points were established based on the labeling of R values proposed by Hinkle et al. [42]. Values of r above 0.9 were labeled as “very highly positive”, between 0.7–0.9 as “highly positive”, between 0.5–0.7 as “moderately positive”, 0.3–0.5 as “low positive”, and between −0.3–0.3 as “little if any correlation”. For statistical analysis, Statistical Package for the Social Sciences (SPSS) version 21.0 for Windows (SPSS Inc., Chicago, IL, USA) was used. The *p*-value criteria were 0.05 as statistical significance.

## 3. Results

Thirty-six workers were considered for this study, all from the same plant of an international manufacturing company, with 6 women and 30 men in the sample. The workers were 40 ± 8 years of age, with a height of 173.2 (7.2) cm and a weight of 78.4 (11.1) kg (Table 1). At the beginning of the workday, one worker reported foot discomfort, and two reported discomfort in the lower back, while none reported knee discomfort. At the end of the workday, six workers reported discomfort in the foot region; two reported discomfort in the knee region, and eight in the lower back region. Differences in weight and height were found between men and women. No differences were observed in any of the foot pressure variables studied, body mass index, or age between men and women.

The *t*-test of repeated measurements found differences between pre- and post-work evaluations in the right foot surface (*p* = 0.02) and total foot surface (*p* = 0.01) (Table 2). In total, right and left foot surface areas increased at the end of the day with a small effect size. Moreover, a significant increase in foot discomfort was found between pre- and post-assessments in the Wilkoxon signed rank test (*p* = 0.02). The increase in lumbar discomfort was 25% and 80% for foot discomfort in reference to the range in the baseline measurement (taken as 100%).

The total foot surface correlated negatively to total mean pressure both at the beginning (r = −0.670, *p* = 0.001) and at the end of the day (r = −0.679, *p* = 0.001). The correlation analysis shows a negatively significant correlation between the increment of the right foot surface during the day and the difference between pre- and post-foot discomfort (r = −0.336, *p* = 0.045). This relationship was not found between the increase in total foot surface and the change in foot discomfort. Furthermore, a correlation has been found between the difference between pre- and post-evaluation in fore mean pressure and the weight of the subjects (r = 0.403, *p* = 0.02).

## 4. Discussion

In this study, the total and right foot surfaces increased during the day (*p* < 0.05). The left foot surface did not increase significantly, but the effect size was similar to the right foot. Also, the discomfort in feet increased after the workday, and a correlation was found between the increase in foot discomfort and the right foot mean pressure change during the day, finding more discomfort in workers who experienced less change in foot mean pressure.

Manufacturing workers who spend more time during the workday in a standing position registered an increase in musculoskeletal discomfort, modifying their posture during a prolonged standing period [37,43].

According to the discomfort registered in the previous workday evaluation, a low value of discomfort in feet and lower back was reported by the workers, with 3.3% and 16.6% of male workers, respectively. Women did not register discomfort in the previous work’s evaluation, which might be due to the reduced sample of women in this study (n = 6). Other studies have found a higher percentage of workers (48%) on assembly lines declaring ankle/foot discomfort or pain [44]. Higher values of work-related pain are found in workers in Denmark; unskilled workers of production companies present a rate of 22% of workers with lower-limb pain and a rate of 25% with lower-back pain [45]. In the present study, the discomfort values in the lower back did not increase significantly over the workday studied. It should not be overlooked that discomfort increased in all three regions, mostly in feet (+1.59 units), followed by lower back (+0.58 units), and knees (+0.24 units). Although the values found in the present study are small, this could be due to the fact that workers selected for this study were considered healthy in the inclusion criteria, where workers with previous sick leave in the last three months were excluded.

In reference to the increase in the intensity of discomfort due to work activity, the lower limb and lower back are the regions with a significant increase in discomfort level, but not in the present study. The trend was an increase in discomfort in the three regions studied. The muscular fatigue accumulated during the workday increases the perception of discomfort of the workers in prolonged standing positions [46]. However, this discomfort was reduced after a rest period of 30 min. The effect on the subjective perception of discomfort could be short-lived and mitigated after a period of rest. Prolonged standing for more than 30 min per hour supposes a higher hazard rate of suffering lower-back pain (2.1) and lower-limb pain (1.7) than in the working population that does not maintain this posture [45]. Standing for a long time of more than 10 min during the day, considering the work and leisure time, has been related to lower-back pain intensity [47]. Venous return could be affected by standing, reducing the blood supply to the lower-limb muscles with the associated fatigue and discomfort. Blood pooling and swelling could be related to the discomfort in the lower limb. In that way, work-related standing may pose a risk of lower-limb vascular problems [10].

Low back discomfort due to prolonged standing could be associated with different possible mechanisms, such as muscular and postural factors and lumbar loading [10,12]. Among the muscular mechanisms, hip kinematics (endurance, co-contraction, and stiffness) could affect the lower-back discomfort level. Foot discomfort could also be influenced by the asymmetrical position of the feet during standing work. This could cause different changes in pressure parameters between the right and left feet or between the dominant and non-dominant feet [36].

Different models have been proposed to predict risk factors of lower-back pain in a standing position, which included, among other factors, the sway in the anteroposterior direction of the center of pressure, the presence of gaps of activation of the gluteus medius, and the degree of L4-L5 axial twist [48]. This study considered 2 h of standing for the measurements. Longer standing time should cause higher levels of lower-back pain.

Referring to the plantar pressure measurements, an increase in foot surface is produced during the workday, being only significant for the right foot. Despite the increase in the left foot (*p* = 0.08) not being significant, the right foot and left foot surfaces increased during the workday by a comparable amount (4.0 and 3.2 cm^2^, over 3%). Postural sway could affect the pressure distribution in the feet [49]. Simon et al. also observed different changes depending on the side of the foot in which the pressure was analyzed after a 3-h day of standing and office work [36]. The findings of the present investigation are in line with this study; the mean and peak foot pressure did not significantly change after the workday [36]. Lower limb fatigue generated by prolonged standing can be due to vascular mechanics, not only muscle fatigue mechanics [12]. In the present study, the increase in right foot mean pressure is correlated to the decrease in the discomfort in feet during the workday. The adaptation of the load distribution could minimize pressure related to foot pain [50]. Increased plantar pressure has been considered a mechanism of foot discomfort [51,52]. Furthermore, pressure deforms the skin, activating the nociceptors and generating a pain sensation [53]. It has been found that a standing position could increase the sensitivity of the foot, lowering the foot pain pressure threshold [54]. The results of the present study do not relate greater plantar pressure to higher discomfort at the end of the workday of assembly-line workers.

A positive correlation has been found between the forefoot mean pressure and the absolute weight of the subjects. Previous research has found a correlation between the body weight and the pressure distribution [55]. A high weight could be a risk factor for foot discomfort caused by standing.

Several strategies have been proposed for reducing the discomfort due to standing as standing aid systems [56], the use of softer flooring and cushioned shoe insoles [57], or declined surfaces. Unstable footwear could be another solution for standing workers to reduce lower-limb discomfort [58], increasing muscular activity that could help venous return. This could be a good solution for leg discomfort treatment. However, on the one hand, the effectiveness of the unstable shoes during a whole workday has not yet been tested. On the other hand, the workers in manufacturing companies must wear safety shoes. So, this solution, although interesting, cannot be applied to this population.

Other proposals focus on the inclusion of short or large breaks during the workday [59], comparing active vs. passive breaks [60]. This kind of solution could be difficult to implement for manufacturing workers where the assembly chain cannot stop. Another option can be for the company to hire extra workers who step in when line workers take their breaks. Other interventions to prevent musculoskeletal discomfort have used exercise and insoles in a work environment. An intervention of 8 weeks of simultaneous use of custom-made insoles and exercises might be an effective intervention to reduce discomfort in the lower limbs and lower back in workers who remain standing for prolonged periods [61].

One of the limitations of this study is that the pressure was measured during a specific moment (pre–post workday). It would be very interesting to record the distribution of foot pressure during the workday. Simon et al. recorded in-shoe plantar pressure for 12 min during a workday [36]. It would be interesting to observe the distribution of the load within the assembly line. The pressure measurement on the platform is carried out in an ideal position (feet in the same position), while standing on the mounting chain may be different. One of the limitations of this study is that the dominant side of the workers was not recorded. These data could help to understand the changes in plantar pressure on one side compared to the other. The sample used in this research does not allow for the results to be statistically extrapolated to other populations. Only six women participated in this study. The difference in weight between men and women may have influenced some results, such as correlations. In the future, it would be necessary to have a greater number of women to establish analyses by groups. It was not considered whether the subjects who experienced discomfort after the workday were related to having correct occupational ergonomics, such as the height of the work tables. This aspect would be interesting to investigate in future studies. Forefoot and rearfoot areas could have been measured more accurately by using the anatomical marks of each subject. The software used did not allow for modifying the selection. All measurements were carried out on Monday without considering weekend activities. This aspect can be considered a possible limitation of this study because the activities could have been different between participants. Misalignments in the foot were not evaluated in the participants.

The research was carried out in the work environment, obtaining values from the workers of an assembly line. Future research would have to clarify the relationship between the change in pressures during the workday and foot discomfort. Future studies could investigate workers with pre-existing musculoskeletal diseases or cardiovascular health conditions to analyze if those workers are especially vulnerable. In turn, the effects of the working day on workers with different foot types (flat feet, for example) should be studied. Future research should focus on evaluating breaks during work and rest cycles as an intervention to act against the appearance of discomfort.

## 5. Conclusions

Prolonged maintained standing during 8 h workday could increase foot discomfort and total foot surface in assembly-line workers. Although the total foot surface increased, the foot mean pressure did not change significantly at the end of work. The pressure in the forefoot and rearfoot areas did not change during the workday. No association was found between the decrease in total foot mean pressure and the increase in foot discomfort after a day of work. The baropodometric measurements (foot surface and pressure) provide information, but in this research, they are not associated with the variables of musculoskeletal discomfort.

## Figures and Tables

**Table 1:** Baseline characteristics of the sample and differences between genders.

	Male (n = 30)	Female (n = 6)	*p*
Variable	Mean (SD)	Mean (SD)	
Age (years)	40 (8)	41 (2)	0.42
Height (cm)	175.2 (5.9)	163.1 (4.4)	0.00 *
Weight (kg)	81.2 (9.4)	64.5 (7.9)	0.00 *
BMI (kg/m^2^)	26.4 (3.2)	24.2 (3.1)	0.31
Variable	Mean (Range)	Mean (Range)	
Foot discomfort (score)	0.06 (2)	0	0.63
Knee discomfort (score)	0	0	1
Lower-Back discomfort (score)	0.26 (6)	0	0.78

* *p* < 0.05. SD: Standard Deviation.

**Table 2:** Pre- and post-8-hour work in standing position baropodometric values and discomfort scores (N = 36).

**Baropodometry Variable**	**PRE-Workday**	**POST-Workday**	**t**	***p***	**Effect Size**	**ES 95% CL**	
						**Lower**	**Upper**
Right foot surface (cm^2^)	107.1 (22.6)	111.1 (25.1)	−2.4	0.02 *	0.400	−0.73	−0.05
Left foot surface (cm^2^)	104.4 (23.5)	107.6 (22.5)	−1.75	0.08	0.292	−0.62	0.04
Total foot surface (cm^2^)	211.5	218.7	−2.60	0.01 *	0.435	−0.77	−0.6
Fore mean pressure (kPa)	242.8 (43.0)	246.5 (53.1)	−0.72	0.42	0.126	−0.46	0.21
Rear mean pressure (kPa)	315.1 (80.4)	303.6 (60.2)	1.1	0.27	0.193	−0.15	0.53
Left mean pressure (kPa)	273.9 (63.1)	266.4 (55.5)	1.17	0.24	0.205	−0.14	0.54
Right mean pressure (kPa)	284.0 (58.2)	283.7 (53.0)	0.04	0.9	0.008	−0.33	0.34
Total foot pressure (kPa)	557.9 (116.7)	550.2 (104.7)	0.76	0.22	0.134	−0.21	0.47
**Discomfort Variable**	**PRE-Workday**	**POST-Workday**	**Z**	***p***			
Feet discomfort (total score)	0.05 (0.33)	1.64 (4.5)	−2.207	0.03 *	-		
Knees discomfort (total score)	0 (0)	0.24 (0.97)	−1.414	0.15	-		
Low Back discomfort (total score)	0.22 (1.04)	0.8 (1.84)	−1.547	0.12	-		

Mean (SD); * *p* < 0.05. Total foot surface (Left and right sum). Fore mean pressure (left and right sum). Rear mean pressure (left and right sum). Total foot pressure (left and right sum). ES: Effect size. CL: Confidence limits.

## Data Availability

The raw data supporting the conclusions of this article will be made available by the authors upon request.
